# Characteristic craniofacial defects associated with a novel *USP9X* truncation mutation

**DOI:** 10.1038/s41439-024-00277-w

**Published:** 2024-05-16

**Authors:** Namiki Nagata, Hiroshi Kurosaka, Kotaro Higashi, Masaya Yamaguchi, Sayuri Yamamoto, Toshihiro Inubushi, Miho Nagata, Yasuki Ishihara, Ayumi Yonei, Yohei Miyashita, Yoshihiro Asano, Norio Sakai, Yasushi Sakata, Shigetada Kawabata, Takashi Yamashiro

**Affiliations:** 1 Department of Orthodontics and Dentofacial Orthopedics, Osaka University Graduate School of Dentistry, Suita, Japan; 2 Department of Microbiology, Osaka University Graduate School of Dentistry, Suita, Japan; 3 Department of Removable Prosthodontics and Gerodontology, Osaka University Graduate School of Dentistry, Suita, Japan; 4 Bioinformatics Research Unit, Osaka University Graduate School of Dentistry, Suita, Japan; 5 Bioinformatics Center, Research Institute for Microbial Diseases, Osaka University, Suita, Japan; 6 Center for Infectious Diseases Education and Research, Osaka University, Suita, Japan; 7 Department of Cardiovascular Medicine, Osaka University Graduate School of Medicine, Suita, Japan; 8 Department of Genetic Counseling, Osaka University Hospital, Osaka, Japan; 9 Child Healthcare and Genetic Science Laboratory, Division of Health Sciences, Osaka University Graduate School of Medicine, Suita, Osaka Japan

**Keywords:** Genetic testing, Mutation

## Abstract

Germline loss-of-function mutations in *USP9X* have been reported to cause a wide spectrum of congenital anomalies. Here, we report a Japanese girl with a novel heterozygous nonsense mutation in *USP9X* who exhibited intellectual disability with characteristic craniofacial abnormalities, including hypotelorism, brachycephaly, hypodontia, micrognathia, severe dental crowding, and an isolated submucous cleft palate. Our findings provide further evidence that disruptions in *USP9X* contribute to a broad range of congenital craniofacial abnormalities.

Ubiquitin signaling plays a wide variety of roles in both embryonic development and general cellular activities; thus, its disruption can result in a wide range of cellular and tissue defects. This signaling pathway is governed by multiple classes of proteins, one of which is deubiquitylases (DUBs), including ubiquitin-specific peptidase 9 X-linked (USP9X)^1^. Germline loss-of-function mutation of *USP9X* has been shown to cause intellectual disability as well as other congenital anomalies. Multiple reports have documented the features of developmental delay resulting from *USP9X* mutations, which is characterized by retardation of neurogenesis, while the etiology of other characteristic features, such as craniofacial anomalies including intraoral features, has not been reported in detail^2^.

The current patient, a 7-year-old Japanese girl, was referred to the Department of Orthodontics at Osaka University Dental Hospital for the correction of malocclusion. She was born at 40 weeks and 6 days gestation, with a weight of 2768 g, and is the second child of healthy, nonconsanguineous parents. Antenatal ultrasound examinations during pregnancy revealed an umbilical artery. Postnatally, she experienced peripheral circulatory failure due to polycythemia. MRI revealed Dandy–Walker syndrome. She exhibited developmental delays, such as poor weight gain, and underwent intubation feeding at 4 months of age. She also exhibited intellectual disability and required assistance for some subjects at school. Her facial features included hypotelorism, a short columella with a wide nasal base, midfacial deficiency with a thin upper lip (Fig. 1a), low-set ears, and micrognathia (Fig. 1b). Intraoral features included a high-arched palate and malocclusion, such as severe crowding on the lower jaw and an underbite (Fig. 1c–e). Clinical manifestations included hypernasality caused by a submucous cleft palate. In addition, an irregular pigment pattern was detected bilaterally on the upper arms (Fig. 1f). Cephalometric analysis revealed a short anteroposterior maxillary diameter and a retruded mandible (Fig. 2a). Hypodontia (involving the upper right first premolar and bilateral upper second premolars) was evident on panoramic radiography (Fig. 2b). A CT scan of the head revealed plagiocephaly without clear evidence of craniosynostosis and an ectopically positioned upper left canine (Fig. 2c).

**Fig. 1:**
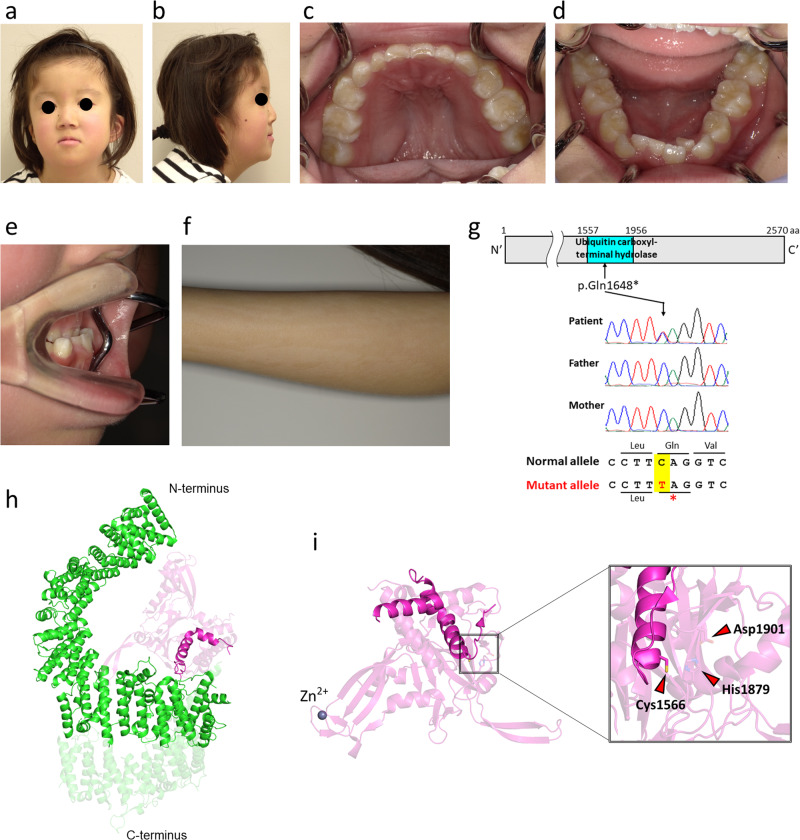
Clinical features of the present patient with a pathogenic mutation in *USP9X.* Frontal (**a**) and lateral (**b**) views of the facial profile. Intraoral photos of the upper (**c**) and lower (**d**) jaws. **e** Underbite was observed in the occlusion. **f** Abnormal pigment pattern on the arm. **g** De novo nonsense mutation in the middle of the ubiquitin carboxyl-terminal hydrolase domain of *USP9X*. **h** The cryo-EM structure of USP9X (PDB ID: 7YXX). The ubiquitin carboxyl-terminal hydrolase domain is shown in cyan. The faded color indicates the truncated area in the mutated protein. **i** The crystal structure of the ubiquitin carboxyl-terminal hydrolase domain (PDB ID: 5WCH) is shown in magenta. His1879 and Asp1901 are missing in the mutated USP9X protein.

**Fig. 2:**
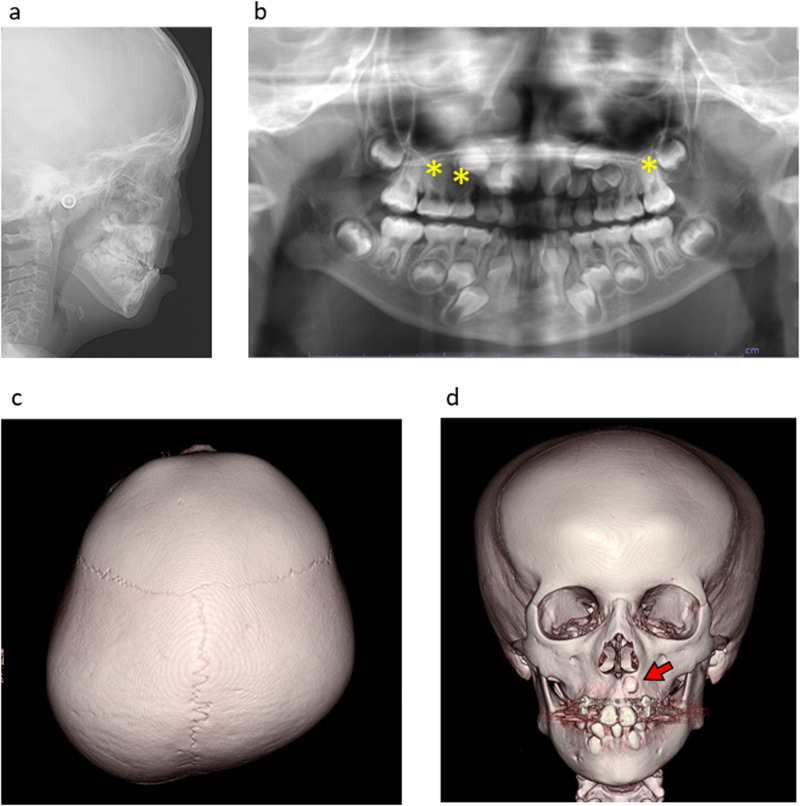
Craniofacial findings from radiographic records. **a** Lateral cephalogram. **b** Orthopantomogram showing congenital missing teeth (yellow asterisk). CT of the head showed plagiocephaly (**c**) and an ectopically positioned left upper canine (**d**, red arrow).

After comprehensive counseling, explaining the risks and benefits of genetic testing, and obtaining informed consent, whole-exome sequencing was performed using genomic DNA extracted from peripheral lymphocytes of the patient and her parents using the Sure Select Human All Exon Kit V6 (Agilent Technologies, Santa Clara, CA, USA). Sequencing was conducted on the NovaSeq 6000 platform (Illumina, San Diego, CA, USA) to elucidate the causative genes for the patient’s phenotypes. A de novo heterozygous nonsense mutation in *USP9X* (NM_001039590, hg19: c.4942 C > T: p.Q1648*), which creates a premature stop codon in the middle of the ubiquitin carboxyl-terminal hydrolase (UCH) domain, was identified (Fig. 1g); this mutation has not been documented in previous studies. As a result of this mutation, USP9X is truncated to 1647 amino acids. The USP domain required for the deubiquitination activity of USP9X has a length of 1557 to 1956 amino acids (Fig. 1g). The mutant form of the *USP9X* protein lacks two crucial amino acid residues within the catalytic triad, strongly suggesting a deficiency in enzymatic activity (Fig. 1h, i)^3^.

These results indicate that the loss of function of *USP9X* during embryonic development could result in multiple craniofacial anomalies. However, the mechanism underlying these craniofacial defects is largely unknown. Embryonic craniofacial development relies on coordinated cellular activities, the failure of which could result in a wide variety of morphological and functional defects. Cranial neural crest cells are among the most important cell populations involved in normal craniofacial development. Multiple genetic mutations associated with the developmental process of cranial neural crest cells result in a wide variety of craniofacial defects, including a characteristic facial appearance and orofacial cleft^4^. Pigment cells are also known to be derived from neural crest cells; therefore, defects in neural crest cells could result in pigment cell defects, as observed in the present case, indicating that neural crest cell defects result from the mutation of *USP9X*.

Mutations in *USP9X* in humans have been demonstrated to induce intellectual disability by influencing neurogenesis through the ubiquitin signaling pathway^1,5^. Interestingly, fibroblasts from patients with *USP9X* mutations have been demonstrated to exhibit a diminished biological response to the TGFβ signaling pathway, as evidenced by reduced signaling in reporter assays and inhibited cell migration^6^. In addition, the TGFβ signaling pathway has been shown to phosphorylate USP9X, stabilizing ankyrin-G through deubiquitination in dendritic spine development^7,8^. TGFβ signaling has also been shown to be critically involved in craniofacial development at various levels. For example, reducing TGFβ signaling in cranial neural crest cells has been demonstrated to increase cell death, subsequently resulting in partial loss of cranial bone^9^. TGFβ signaling plays critical roles in palatogenesis; loss of function results in cleft palate in both mice and humans^10,11^. In addition, USP9X has been shown to deubiquitylate DVL2, another key protein regulating the Wnt signaling pathway, altering its activity in the cell^12^. Canonical Wnt signaling is another central pathway involved in normal craniofacial development. Eliminating beta-catenin, a mediator of canonical Wnt signaling, from neural crest cells causes severe craniofacial development in mice^13^. Interestingly, polymorphisms in Dvl2 have been associated with susceptibility to orofacial clefts in the Polish population^14^. Canonical Wnt signaling is also known to regulate the development of dentition, and gene mutations in this pathway could cause the loss of permanent teeth, as in the present case^15^. Moreover, it has been demonstrated that USP9X is involved in ciliogenesis through the regulation of ubiquitination of key ciliogenic proteins^16^. Primary cilia serve as sensors for cells, transducing multiple signaling pathways that include critical molecules for embryonic craniofacial development^17^. The disease spectrum resulting from defects in primary cilia is called ciliopathy, which is associated with a wide variety of congenital defects. Notably, up to 30% of ciliopathies can be primarily defined by craniofacial phenotypes, clearly indicating a biological connection between ciliogenesis and craniofacial development. Craniofacial manifestations of ciliopathy include several phenotypes exhibited in the present case, such as orofacial cleft, facial midline defects such as hypotelorism, micrognathia, and Dandy–Walker malformation, which together are found in the majority of ciliopathy patients^18^. Considering that the present patient exhibited polydactyly and intellectual disability, which are among the core phenotypic features of ciliopathy, the loss of function of USP9X may be mechanistically related to abnormal ciliogenesis, but further studies are needed to confirm this supposition.

Pathogenic mutations in *USP9X* are known to exhibit a wide variety of phenotypes. Although no clear genotype‒phenotype correlation has been reported, nonsense-mediated mRNA decay and differential X-chromosome inactivation (XCI) are potential mechanisms that could affect the pathogenesis and phenotypic diversity. Notably, *USP9X* has been shown to escape XCI, which could influence the phenotype of females^19^. Studies have highlighted the differences between sexes, with females often exhibiting strong loss-of-function mutations such as premature termination codons, while males tend to have milder forms, such as missense mutations^5,6^.

## HGV database

The relevant data from this Data Report are hosted at the Human Genome Variation Database at 10.6084/m9.figshare.hgv.3402.
